# Mosquito coil exposure associated with small cell lung cancer: A report of three cases

**DOI:** 10.3892/ol.2015.2922

**Published:** 2015-02-02

**Authors:** JIE ZHANG, HUI-WEI QI, YU-PING SUN, HUI-KANG XIE, CAI-CUN ZHOU

**Affiliations:** 1 Department of Oncology, Shanghai Pulmonary Hospital, Tongji University School of Medicine, Shanghai 200433, P.R. China; 2 Department of Pathology, Shanghai Pulmonary Hospital, Tongji University School of Medicine, Shanghai 200433, P.R. China

**Keywords:** insecticide, lung carcinogen, octachlorodipropyl ether, occupational exposure

## Abstract

Mosquito coils, which are commonly used as residential insecticides in Asia, contain different concentrations of octachlorodipropyl ether (S-2) as a synergist or an active ingredient. As bis(chloromethyl) ether (BCME) is an extremely potent lung carcinogen that can be produced by the thermolytic degradation of S-2, contact with mosquito coils is likely to expose individuals to a certain level of BCME, and therefore increase the risk of lung cancer. However, the significance of exposure is uncertain, as clinical and epidemiological studies concerning mosquito coil users and workers are lacking. The present study describes three cases of small cell lung cancer treated at the Shanghai Pulmonary Hospital that were likely to be the result of exposure to mosquito coils. All patients had worked in the mosquito coil manufacturing industry, with an mean occupational duration of 9.1 years, and presented with similar respiratory symptoms, such as cough and dyspnea. Upon diagnosis, no metastasis to other organs was identified in any of the cases. Subsequently, the three patients were treated with chemotherapy as well as radiotherapy in one case, however, all patients succumbed to the disease, with a mean overall survival time of 10.7 months. We conclude that contact with mosquito coils is likely to expose individuals to a level of S-2 that may increase the risk of SCLC.

## Introduction

Small cell lung cancer (SCLC) accounts for ~15% of all lung cancers in the USA (1). The majority of patients typically present with symptoms associated with central airway disease, such as cough and dyspnea, as well as symptoms of widespread metastatic disease, such as weight loss, debility, bone pain and neurologic compromise. Histological analysis may be sufficient for the diagnosis of SCLC, which presents as poorly differentiated tumor that is categorized as a high-grade neuroendocrine carcinoma. The majority of SCLCs are immunoreactive for keratin and thyroid transcription factor-1 (TTF-1). Furthermore, at least one marker of neuroendocrine differentiation, such as neuron-specific enolase (NSE), neural cell adhesion molecule (CD56) or synaptophysin (2), is observed in 75% of SCLC cases (2,3). Despite its rapid doubling time and early development of widespread metastases, SCLC is highly sensitive to chemotherapy and radiotherapy (4). At present, the etoposide and cisplatin regimen is the standard first-line chemotherapy treatment for patients with SCLC. However, the majority of patients exhibit recurrences and subsequently succumb to the disease (5,6). Patients with SCLC are usually classified as either limited-or extensive-stage. Limited-stage SCLC is defined as cancer within only one lung and/or in the lymph nodes in the mediastinum. The majority of patients with SCLC are extensive-stage at the time of initial diagnosis, where the cancer has spread either to the other side of the chest or to more distant locations in the body. The median survival rates are 10.3 months and 5.6 months for patients with limited- and extensive-stage disease, respectively (7).

Smoking is, undoubtedly, the primary cause of lung cancer (8,9). Environmental and occupational exposures are also potential risk factors, but the effects of these suspected factors remain equivocal (10,11). Mosquito coils are a major type of residential insecticide, and are frequently burned indoors and outdoors in Asia. In China, mosquito coils that contain pyrethroid insecticides, particularly d-allethrin, use different concentrations of octachlorodipropyl ether (S-2) as a synergist or an active ingredient (12). S-2 has been reported to exhibit carcinogenicity following its degradation to the extremely potent lung carcinogen, bis(chloromethyl) ether (BCME) (13). The carcinogenicity of BCME was first demonstrated in 1968 with skin painting in mice and subcutaneous injection in rats (14), subsequently a number of additional animal experiments have been performed. Van Duuren *et al* (14) stated that BCME produced a moderate tumor response as an initiator and caused hyperplasia in the treated area. Preceding the increase in DNA synthesis, an inhibition of DNA synthesis immediately followed treatment with BCME. The reduction in apparent DNA synthesis caused by the agent may occur via a number of mechanisms (15), including: i) Interference with strand separation and/or DNA polymerase activity due to the presence of a covalently bound foreign molecule in DNA, ii) reduction in the number of cells synthesizing DNA due to of cell death or other toxic reactions, iii) breakdown of (labeled) DNA following treatment, and iv) defective enzymes resulting in a failure to utilize nucleoside precursors. Furthermore, BCME is a unique alkylating agent due to the participation of the oxonium ion in addition to the carbonium ion usually encountered with alkylating agent carcinogens. The existence of this equilibrium reaction and the resonance stabilization in the ionic species explains the induction of lung cancer in animals and humans by this carcinogen and its potential for resulting in the induction of malignant tumors at distant sites (16). Exposure to BCME via inhalation has been associated with the formation of lung tumors in rats and mice (17–19). Furthermore, previous studies have shown that BCME leads to mutations in bacteria, as well as unscheduled DNA synthesis in cultured human cells (15,20). In addition, epidemiological studies from various geographical locations, including the USA (21), Germany (22) and Japan (23) have found that occupational exposure to BCME is associated with the development of lung cancer, in particular SCLC (16,20). The present study describes three cases of small cell lung cancer (SCLC) that were likely to have arisen due to exposure to mosquito coils. Consent was obtained from the families of the patients.

## Case reports

### Case one

**Figure 1:**
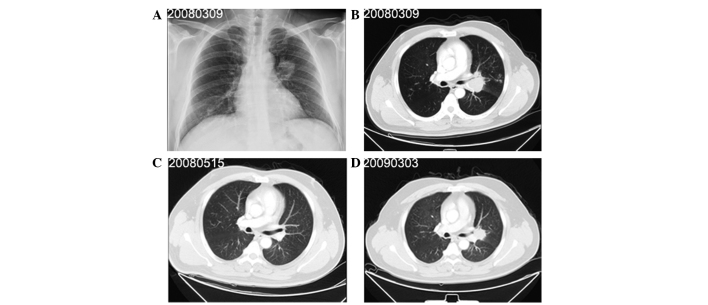
Case one: Representative images from radiography and chest CT revealing the presence of a mass in the upper lobe of the left lung. (A) Radiograph prior to treatment. (B) Representative CT prior to treatment. (C) Representative CT image after two cycles of first-line chemotherapy. (D) Representative CT image showing progressive disease after six cycles of first-line chemotherapy. CT, computed tomography.

A 39-year-old male never-smoker presented to the Shanghai Pulmonary Hospital (Tongji University, School of Medicine, Shanghai, China) on March 6, 2008, with a productive cough that had been apparent for one month. Radiography (Fig. 1A) and computed tomography (CT) of the chest (Fig. 1B) revealed enlarged lymph nodes and a mass measuring 4.8×3.4 cm in the upper lobe of the left lung. Immunohistochemical analysis indicated that the tumor was positive for thyroid transcription factor 1 (TTF-1) and synaptophysin (SYN), but negative for cluster of differentiation (CD)5 and 6 (Fig. 4). The patient was subsequently diagnosed with SCLC, tumor-node-metastasis (TNM) stage T4N2M0 (IIIb). Following two cycles of chemotherapy with 100 mg/m^2^ etoposide and 75 mg/m^2^ cisplatin on days one to three of three-weekly cycles, the patient exhibited a complete response (CR) (Fig. 1C). The six cycles of chemotherapy were completed on September 13, 2008. In March 2009, CT revealed the presence of progressive disease (PD) (Fig. 1D) and second-line chemotherapy with 60 mg/m^2^ irinotecan on days one and eight of three-weekly cycles, was subsequently initiated. Due to a poor performance status, the patient proceeded to receive supportive care, but succumbed to the disease on August 17, 2009.

### Case two

**Figure 2:**
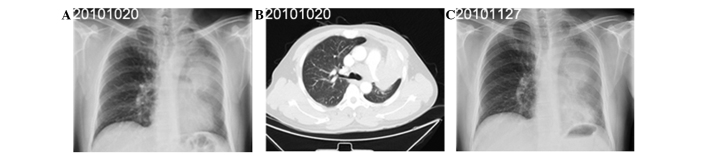
Case two: Representative images from radiography and chest CT revealing the presence of a mass in the upper lobe of the left lung and enlarged lymph nodes. (A) Radiograph prior to treatment. (B) Representative CT image prior to treatment. (C) Radiograph after one cycle of first-line chemotherapy treatment. CT, computed tomography.

A 41-year-old male presented to the Shanghai Pulmonary Hospital on October 20, 2010, with a productive cough and dyspnea. The patient had smoked 10 cigarettes per day for the past 20 years. Radiography (Fig. 2A and C) and CT (Fig. 2B) of the chest revealed enlarged lymph nodes and a mass measuring 10.5×7.2 cm in the upper lobe of the left lung. Immunohistochemical analysis indicated that the tumor was positive for TTF-1 and SYN, but negative for CD5/6 (Fig. 4). The patient was subsequently diagnosed with SCLC, stage T4N2M0 (IIIb). Following two cycles of chemotherapy with 100 mg/m^2^ etoposide and 25 mg/m^2^ cisplatin on days one to three of three-weekly cycles, the patient’s condition deteriorated, with evidence of hemoptysis and thrombocytopenia. The patient succumbed to the disease on January 25, 2011.

### Case three

**Figure 3:**
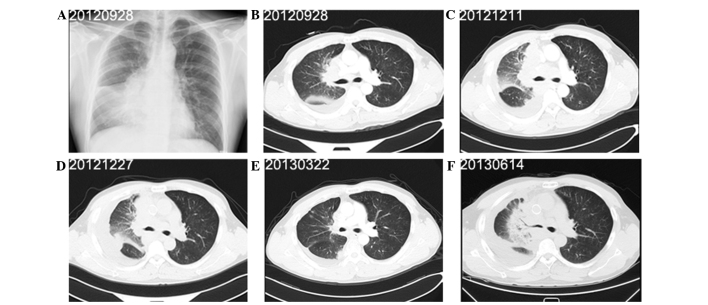
Case three: Representative images from radiography and chest CT revealing the presence of a mass in the middle lobe of the right lung, pleural effusion and enlarged lymph nodes. (A) Radiograph prior to treatment. (B) Representative CT image prior to treatment. (C) Representative CT image after two cycles of first-line chemotherapy. (D) Representative CT image after superior vena cava stenting. (E) Representative CT image after thoracic radiation therapy and four cycles of second-line chemotherapy. (F) Representative CT image showing progressive disease after second-line chemotherapy. CT, computed tomography.

**Figure 4:**
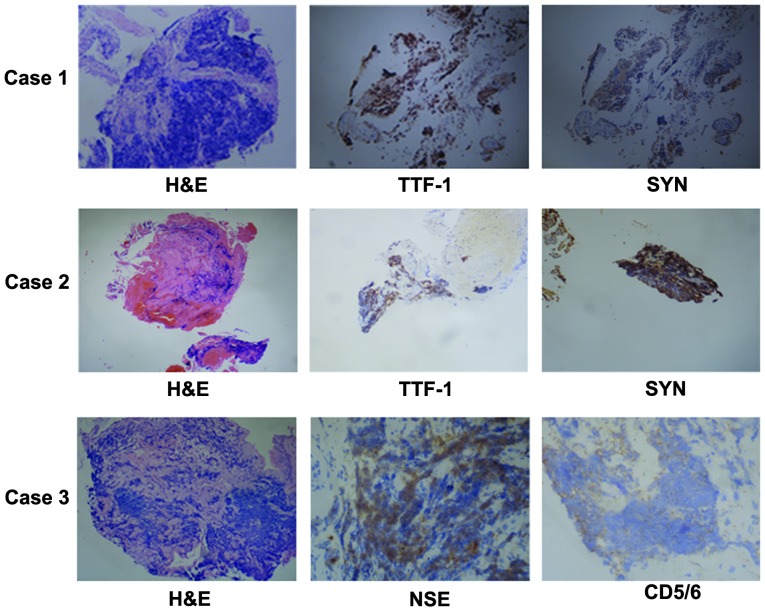
Histological analysis of endobronchial biopsy specimens from cases one, two and three (magnification, ×100). H&E, hematoxylin and eosin; TTF-1, thyroid transcription factor 1; SYN, synaptophysin; NSE, neuron-specific enolase; CD, cluster of differentiation.

A 40-year-old male presented to the Shanghai Pulmonary Hospital on September 27, 2012, with right-sided chest pain, a productive cough and dyspnoea that had been apparent for two weeks. The patient had smoked 15 cigarettes per day for the past 18 years. Radiography (Fig. 3A) and CT of the chest (Fig. 3B) revealed pleural effusion, enlarged lymph nodes and a mass measuring 9.4×8.0 cm in the middle lobe of the right lung. Immunohistochemical analysis indicated that the tumor was positive for TTF-1, NSE, chromogranin A and Ki-67, but negative for SYN, leukocyte common antigen, p63 and CD5/6 (Fig. 4). The patient was subsequently diagnosed with SCLC, stage T4N3M1a (IV). Following two cycles of chemotherapy with 100 mg/m^2^ etoposide and 25 mg/m^2^ cisplatin on days one to three of three-weekly cycles, the tumor response was assessed as PD (Fig. 3C). Superior vena cava stenting and 25 Gy thoracic radiation therapy (2.5 Gy/fraction) were performed in December, 2012 for two weeks. Four cycles of second-line chemotherapy with 60 mg/m^2^ on days one and eight of three-weekly cycles were also administered (Fig. 3E). On June 14, 2013, the tumor response was evaluated as PD (Fig. 3F), at which time the patient’s performance status deteriorated. The patient succumbed to the disease on July 02, 2013.

## Discussion

In China, the burning of mosquito coils is a common practice, particularly in summer. At present, a limited number of toxicity studies have been published that describe the potential carcinogenic effects associated with exposure to coil-containing toxins. A previous case-control study (24) investigated whether exposure to mosquito coil smoke was a risk factor for the development of lung cancer. The results of the study revealed that mosquito coil smoke exposure was more commonly observed in lung cancer patients than in control subjects (38.1 vs. 17.8%; P<0.01), and that the risk of lung cancer was significantly increased among those who frequently burned mosquito coils compared with non-burners (adjusted odds ratio=3.78; 95% CI, 1.55–6.90) (18). Another study demonstrated a positive association between daily exposure to mosquito coils and lung cancer among smokers in China (25). Therefore, exposure to mosquito coil smoke may have a significant role in the pathogenesis of lung cancer.

The key to recognizing cases of lung cancer that are a result of occupational or environmental exposures is clinical investigation and consideration of all possible causes for the disease that are present. The histological type of occupational-induced lung cancers is usually different to that of lung cancers caused by another factor due to the difference in the etiology of lung cancer. Between 2008 and 2012, three patients were diagnosed with SCLC at the Shanghai Pulmonary Hospital. These patients, whose cases have been reported in the present study, were aged around 40 years old, which is younger than the general age of patients with lung cancer. Notably, all patients had been employed in the manufacture of mosquito coils in Xinghua, Jiangsu, for a mean of 9.1 years. The mean overall survival time after presentation was 10.7 months. The primary routes of occupational exposure to mosquito coil toxins are inhalation and dermal contact. Based upon the clinical and pathological findings, it was hypothesized that occupational exposure to mosquito coils was a major factor involved in the pathogenesis of the lung cancer.

Mosquito coils generally consist of an insecticide, a binder, organic fillers capable of smoldering and additives, such as synergists. It is reported that the majority of mosquito coils used in China contain different concentrations of S-2 (26). Since mosquito coils that contain S-2 are unregistered and illegal for use in the United States (10), global studies regarding the effects of S-2 are limited. Exposure to S-2 by inhalation results in oxidative damage to the liver, spleen and lungs of mice. The lung, however, is believed to be the main target organ (27). Since BCME can be produced from impurities contained in S-2, or by the thermolytic degradation of S-2, the use of S-2-containing mosquito coils has been established to be a potential contributor to environmental exposure to BCME (13). The risk of lung cancer increases with increased exposure duration or cumulative exposure (22). A previous study revealed that for workers exposed to the highest doses, the relative risk of developing lung cancer increased more than 10 fold (28). A further study revealed that the mean time between first exposure and diagnosis was 13 years, and that the average age of exposed individuals at diagnosis was 10.5 years lower than of non-exposed individuals (29).

Histological evaluation has indicated that exposure to S-2 results primarily in small cell-type lung cancers, particularly oat-cell carcinoma (29,30). Based upon the histological evidence, it was hypothesized that the inhalation of S-2 may have been the potential cause of SCLC in the three patients included in the present study. However, the other toxic products released following mosquito coil use have yet to be adequately assessed, therefore, future controlled studies should be conducted in order to evaluate their effects. Exposure is a controllable factor, and workers therefore deserve preventive actions in order to reduce exposure to toxins in the workplace. Furthermore, the effects of daily use and exposure to mosquito coils should be evaluated with respect to further health implications.
